# Diabetes duration and types of diabetes treatment in data-driven clusters of patients with diabetes

**DOI:** 10.3389/fendo.2022.994836

**Published:** 2022-11-15

**Authors:** Jie Zhang, Yuanyuan Deng, Yang Wan, Jiao Wang, Jixiong Xu

**Affiliations:** ^1^ Department of Endocrinology and Metabolism, First Affiliated Hospital of Nanchang University, Nanchang, Jiangxi, China; ^2^ Jiangxi Clinical Research Center for Endocrine and Metabolic Disease, Nanchang, Jiangxi, China; ^3^ Jiangxi Branch of National Clinical Research Center for Metabolic Disease, Nanchang, Jiangxi, China

**Keywords:** diabetes, classification of diabetes, cluster analysis, diabetes therapy, duration of diabetes

## Abstract

**Background:**

This study aimed to cluster patients with diabetes and explore the association between duration of diabetes and diabetes treatment choices in each cluster.

**Methods:**

A Two-Step cluster analysis was performed on 1332 Chinese patients with diabetes based on six parameters (glutamate decarboxylase antibodies, age at disease onset, body mass index, glycosylated hemoglobin, homeostatic model assessment 2 to estimate β-cell function and insulin resistance). Associations between the duration of diabetes and diabetes treatment choices in each cluster of patients were analyzed using Kaplan-Meier survival curves and logistic regression models.

**Results:**

The following five replicable clusters were identified: severe autoimmune diabetes (SAID), severe insulin-deficient diabetes (SIDD), severe insulin-resistant diabetes (SIRD), mild obesity-related diabetes (MOD), and mild age-related diabetes (MARD). There were significant differences in blood pressure, blood lipids, and diabetes-related complications among the clusters (all *P* < 0.05). Early in the course of disease (≤5 years), compared with the other subgroups, the SIRD, MOD, and MARD populations were more likely to receive non-insulin hypoglycemic agents for glycemic control. Among the non-insulin hypoglycemic drug options, SIRD had higher rates of receiving metformin, alpha-glucosidase inhibitor (AGI), and glucagon-like peptide-1 drug; the MOD and MARD groups both received metformin, AGI and sodium-glucose cotransporter 2 inhibitor (SGLT-2i) drug ratio was higher. While the SAID and SIDD groups were more inclined to receive insulin therapy than the other subgroups, with SAID being more pronounced. With prolonged disease course (>5 years), only the MOD group was able to accept non-insulin hypoglycemic drugs to control the blood sugar levels, and most of them are still treated with metformin, AGI, and SGLT-2i drugs. While the other four groups required insulin therapy, with SIDD being the most pronounced.

**Conclusions:**

Clustering of patients with diabetes with a data-driven approach yields consistent results. Each diabetes cluster has significantly different disease characteristics and risk of diabetes complications. With the development of the disease course, each cluster receives different hypoglycemic treatments.

## Introduction

Currently, diabetes mellitus is mainly classified in clinical work into type 1 diabetes and type 2 diabetes (1). There is a high degree of heterogeneity in clinical manifestations, comorbidities, and treatment among patients with diabetes, especially type 2 diabetes (2, 3), which hinders accurate diagnosis and treatment. Therefore, better characterizing, understanding, and exploiting the DM heterogeneity may be useful for improving the care and prognosis of patients with DM.

In 2018, Ahlqvist et al. (4) subdivided diabetes into five subtypes based on six indicators [glutamate decarboxylase antibodies (GADA), age at disease onset, body mass index (BMI), glycosylated hemoglobin (HbA1c), homeostatic model assessment 2 to estimate β-cell function (HOMA2-B) and insulin resistance (HOMA2-IR)] of patients with diabetes. The first subtype is defined by the positivity of GADA [severe autoimmune diabetes (SAID)]. The following four type 2 diabetes subtypes are described according to the different degrees of differences in the other five variables: severe insulin-deficient diabetes (SIDD), severe insulin-resistant diabetes (SIRD), mild obesity-related diabetes (MOD), and mild age-related diabetes (MARD). The other researchers have since come up with similar clustering results by analyzing different populations based on the same clustering algorithm (5–7), suggesting that this clustering is reproducible across different populations.

Ahlqvist et al. (4) also reported significant differences in the risk of diabetic complications among the five clusters, and this result was replicated in Chinese (5), Japanese (6), and German (7). However, few studies have comprehensively examined how these five DM clusters are treated in the actual clinical setting. Ahlqvist et al. (4) only mentioned the differences in metformin and insulin use during follow-up in five clusters of patients with new-onset diabetes, and they found that patients with SIRD, MOD, and MARD were less likely to receive insulin treatment than patients with SAID and SIDD. The SIDD group had the highest proportion of metformin use. Lin Xing et al. (8) only analyzed the differences in the use of hypoglycemic drugs among the clusters, but the difference was not assessed in relation to the course of diabetes. They found that the SIDD, MOD, and MARD groups had the highest proportion of insulin treatment, metformin and glucagon-like peptide-1 receptor agonists (GLP-1RA) treatment, and dipeptidyl peptidase 4 inhibitor (DPP-4i) treatment, respectively. In order to improve the medical management of diabetes and promote population health, the American Diabetes Association (ADA) updates its diabetes guidelines with the times, as does the Chinese Diabetes Association. In Chinese diabetes guidelines (9), metformin is the first-line hypoglycemic agent for patients with type 2 diabetes, and sulphonylurea (SU), glinides, α-glucosidase inhibitor (AGI), thiazolidinediones (TZDs), DPP-4i, sodium-glucose co-transporter 2 inhibitors (SGLT2i), GLP-1RA, and insulin are the main combination agents. Through the above-mentioned studies we found differences in the treatment of each DM cluster, that is, diabetic patients with different pathophysiology need to choose different hypoglycemic regimens. In addition, in clinical work, we can find that as the course of diabetes progresses, the appropriate glucose-lowering regimen for the patient changes. Therefore, it is crucial to clarify the diabetes treatment method of each DM cluster with the progression of the disease duration for clinicians to accurately treat diabetes in the future.

The purpose of clustering patients with DM is to ensure that they receive better management and clinical treatment. This study aimed to perform a cluster analysis on Chinese patients with DM according to their clinical indicators and analyze the association between the duration of diabetes and diabetes treatment choices of patients in each diabetes cluster, so as to provide a reference for clinicians to treat patients with each diabetes cluster.

## Materials and methods

### Study population

**Figure 1:**
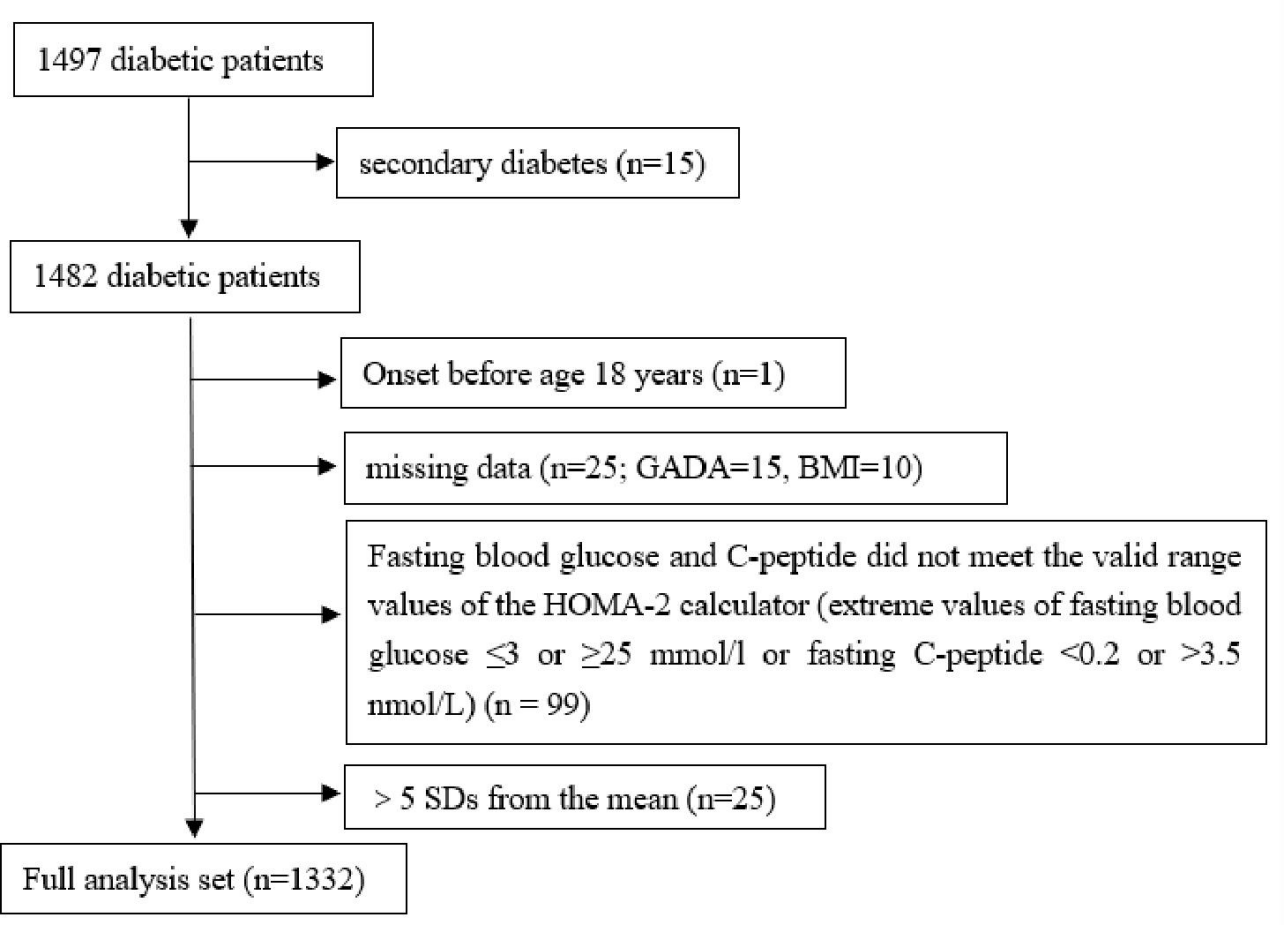
Flowchart of study participants. GADA, glutamate decarboxylase antbody; BMI, body mass index; HOMA, homeostatic model assesment; SD, standard deviation.

This retrospective study investigated a total of 1497 patients with diabetes hospitalized at the Endocrinology Department of the First Affiliated Hospital of Nanchang University from December 2020 to March 2022 and whose blood glucose control was up to standard at the time of discharge. All medical information was collected from electronic medical records by trained medical staff, such as diabetes duration (the time between diabetes diagnosis and hospital admission), diabetes history, family history, antidiabetic drug prescription at discharge, and blood and urine test results. Diabetes was defined by the following diagnostic criteria: fasting plasma glucose (FPG) ≥7.0 mmol/L, 2-h plasma glucose (2hPG) ≥11.1 mmol/L, and/or glycosylated hemoglobin (HbA1c) ≥6.5%. The definition of blood sugar control compliance refers to the standards of medical care for type 2 diabetes in China 2019 (9). In addition, patients with secondary diabetes (such as pancreatic diabetes, drug-induced diabetes; n = 15); patients with diabetes onset before 18 years old (n = 1); patients with missing GADA and BMI data (n = 25); those whose FPG and C-peptide levels do not meet the valid range values of the HOMA2 calculator (10) (extreme value of FPG ≤3 or ≥25 mmol/l or fasting C-peptide level <0.2 or >3.5 nmol/L) (n = 99); and patients with extreme outliers (>5 SDs from the mean; n = 25) were excluded. Overall, 1332 patients with diabetes were included in the study (**Figure 1**). The study protocol was approved by the Ethics Committee of the First Affiliated Hospital of Nanchang University, and the study was conducted in accordance with the Declaration of Helsinki (Ethics No. 2022-4-025).

### Measurements

Participants’ height and weight were measured using an automated anthropometric device, and BMI is calculated by dividing the weight (in kilograms) by the square of the height (in meters). Blood pressure was measured by a trained nurse with a sphygmomanometer.

All patients underwent blood drawing after at least 8 h of fasting. Biochemical parameters, such as FPG, total cholesterol (TC), triglycerides (TG), low-density lipoprotein cholesterol (LDL-c) and high-density lipoprotein cholesterol (HDL-c), were determined by using an automatic analyzer (Roche, Basel, Switzerland). HbA1c was measured using high performance liquid chromatography (Ultra2 HbA1c Detector; Primus Corporation, Atlanta, GA, USA). Urinary albumin (immunological turbidimetry assay) and urinary creatinine (picric acid method) were measured by Biosystems A25. The estimated glomerular filtration rate (eGFR) was calculated using the Chronic Kidney Disease Epidemiology Collaboration equation (11). GADA is measured by using a radioligand assay. The analysis of fasting C-peptide was performed by standard methods, and HOMA2-B and HOMA2-IR were calculated based on FPG and fasting C-peptide levels using the HOMA2 calculator (10).

### Definitions of diabetes and diabetic complications

Type 1 diabetes was defined as GADA-positive with C-peptide level of <0.3 nmol/L. Latent autoimmune diabetes in adults (LADA) was defined as GADA positivity with C-peptide level of ≥0.3 nmol/L. Diabetic kidney disease (DKD) was defined as eGFR <60 ml/min/1.73 m^2^ or UACR ≥30 mg/g. Diabetic retinopathy (DR) is diagnosed by an ophthalmologist based on a fundus examination. Diabetic peripheral neuropathy (DPN) is diagnosed by clinical symptoms, neurological and electrophysiological examinations.

Hypertension was defined as systolic blood pressure (SBP) ≥140 mmHg, diastolic blood pressure (DBP) ≥90 mmHg, or taking an antihypertensive medication. Coronary heart disease (CHD) was defined as a history of angina and/or myocardial infarction. The definition of cerebrovascular disease (CVD) refers to cerebral dysfunction caused by CVD such as cerebral hemorrhage and cerebral infarction.

### Cluster analysis

We employed a Two-Step clustering method among 1332 participants with diabetes. Two-step clustering is an intelligent clustering method in which categorical and continuous variables can be simultaneously addressed, and in which the optimal clustering number is automatically determined. First, we estimated the appropriate number of clusters by the silhouette width method. We then performed a hierarchical cluster analysis as the second step. Hierarchical clustering was carried out for 2-15 clusters using log-likelihood as a distance measure and Schwarz’ s Bayesian criterion for clustering.

### Statistical analysis

Non-normally distributed continuous data were expressed as medians (interquartile range, IQR), and comparisons of continuous variables between groups were performed by the Kruskal-Wallis test. Categorical variables were presented as numbers and percentages, and between-group differences were assessed by R×C crosstab chi-square test or Fisher’s test. Univariate survival analysis was performed using the Kaplan-Meier method, and the results were analyzed using the log-rank test. Logistic regression analysis was used to estimate odds ratios [OR; 95% confidence interval (CI)] for each diabetes cluster with diabetes treatment. The following three statistical models were used: Model 1 did not adjust for covariates; Model 2 adjusted for age and sex; Model 3 was adjusted for age, sex, duration of diabetes, family history of diabetes, smoking, drinking, SBP, DBP, DR, DPN, DKD, CVD, CHD, and hypertension. All data were analyzed using SPSS software (version 26.0, SPSS Chicago, IL, USA), and *P* < 0.05 was considered statistically significant. Box diagram and Kaplan-Meier curve were drawn by Graphpad Prism 8.0 (Graphpad Software Company, CA, USA).

## Results

### Cluster distribution and characteristics at baseline

**Figure 2:**
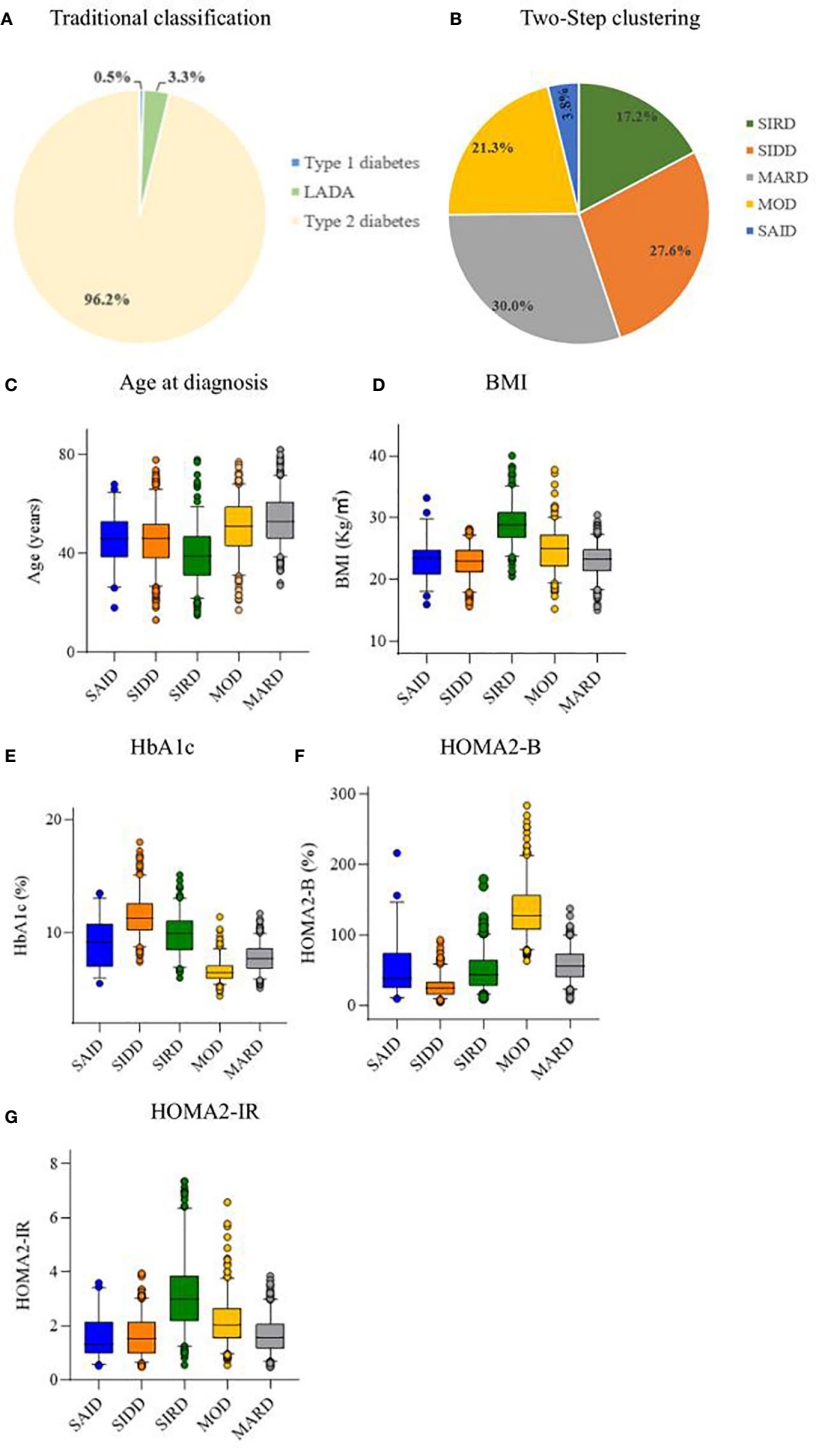
Distribution and characteristics of studied patients. **(A)** Distribution of studied patients according to traditional classification. **(B)** Distribution of study patients according to Two-Step clustering. Characteristics of **(C)** age at diagnosis, **(D)** body mass index (BMI), **(E)** hemoglobin Alc (HbAlc), **(F)** homeostatic model assessment 2 estimates of β-cell function (HOMA2-B), and **(G)** homeostatic model assessment 2 estimates of insulin resistance (HOMA2-IR) for each cluster. LADA, adult latent autoimmune diabetes; SAID, severe autoimmune diabetes; SIDD, severe insulin-deficient diabetes; SIRD, severe insulin-resistant diabetes; MOD, mild obesity-related diabetes; MARD, mild age-related diabetes.

Using the Two-Step cluster analysis, five subgroups were identified. Among the 1332 patients with diabetes, 7 (0.5%) had TIDM, 44 (3.3%) had LADA, and 1281 (96.2%) had type 2 diabetes (**Figure 2A**). The characteristics of the five DM clusters are similar to those reported by Ahlqvist et al. (4). Thus, we gave the five clusters the same class name: cluster 1, SAID; cluster 2, SIDD; cluster 3, SIRD; cluster 4, MOD; and cluster 5, MARD (**Figure 2B**). The cluster centers are shown in **Table S1**.

The SAID group included 51 (3.8%) patients, who were mainly GADA positive, with lower age at diagnosis and BMI, and insulin deficiency. The SIDD group included 368 (27.6%) patients with the lowest HOMA2-B level and high HbA1c level. The SIRD group included 229 (17.2%) patients characterized by the highest HOMA2-IR, high BMI, and young age at diagnosis. The MOD group included 284 (21.3%) patients with high BMI, HOMA2-B, and HOMA2-IR. The MARD group included 400 (30.0%) patients, the oldest group of patients and the most common of the five clusters (**Figure 2**).

### Comparison of clinical characteristics of clusters

The clinical data of the five DM clusters are listed in **Table 1**. Among the basic characteristics, compared with other clusters, SIRD had the highest rates of males (79.5%), smoking (31.0%), and drinking (24.5%), and the lowest duration of diabetes. There was no significant difference in the family history of diabetes among the groups (*P* = 0.371).

**Table 1:** Baseline characteristics of clustering-based subgroups.

	SAID	SIDD	SIRD	MOD	MARD	*P*
n (%)	51 (3.8)	368 (27.6)	229 (17.2)	284 (21.3)	400 (30.0)	
Male, n (%)	30 (58.8)	242 (65.8)	182 (79.5)	165 (58.1)	210 (52.5)	<0.001
Age (years)	56 (46-64)	52 (44-61)	44 (36-55)	58 (49-67)	63 (54-69)	<0.001
Age at Diagnosis (years)	46 (39-53)	46 (38-52)	39 (31-47)	51 (43-59)	53 (46-61)	<0.001
Diabetes Duration (years)	7 (3-12)	5 (0-10)	3 (1-10)	5 (2-10)	8 (2-12)	<0.001
BMI (kg/m^2^)	23.5 (20.8-24.8)	23.0 (21.1-24.8)	28.9 (26.7-30.9)	25.0 (22.0-27.2)	23.2 (21.3-24.9)	<0.001
SBP (mmHg)	130 (113-141)	128 (118-141)	131 (121-146)	131 (118-144)	133 (118-145)	0.108
DBP (mmHg)	75 (68-82)	77 (69-85)	80 (73-89)	73 (66-81)	73 (64-82)	<0.001
Smoking, n (%)	11 (21.6)	105 (28.5)	71 (31.0)	62 (21.8)	85 (21.3)	0.020
Drinking, n (%)	8 (15.7)	84 (22.8)	56 (24.5)	41 (14.4)	73 (18.3)	0.021
Family History of Diabetes, n (%)	17 (33.3)	122 (33.2)	66 (28.8)	82 (28.9)	107 (26.8)	0.371
FPG (mmol/L)	8.3 (6.4-11.5)	11.3 (9.2-13.9)	10.7 (8.8-14.0)	5.4 (4.7-6.2)	7.5 (6.5-8.9)	<0.001
HbA1c (%)	9.1 (7.0-10.8)	11.3 (10.2-12.6)	9.9 (8.5-11.1)	6.5 (5.9-7.1)	7.7 (6.8-8.6)	<0.001
HOMA2-B	37.7 (24.8-73.8)	24.0 (15.7-33.9)	43.3 (28.1-64.5)	127.1 (107.2-156.8)	56.1 (39.8-73.8)	<0.001
HOMA2-IR	1.32 (0.99-2.15)	1.52 (0.97-2.14)	2.98 (2.18-3.85)	2.04 (1.53-2.65)	1.55 (1.16-2.07)	<0.001
LDL-c (mmol/L)	2.3 (1.6-3.2)	2.8 (2.1-3.5)	2.7 (2.0-3.3)	2.4 (1.9-3.0)	2.5 (1.8-3.1)	<0.001
HDL-c (mmol/L)	1.2 (1.0-1.4)	1.1 (0.9-1.3)	0.9 (0.8-1.1)	1.1 (0.9-1.3)	1.1 (1.0-1.3)	<0.001
TC (mmol/L)	4.4 (3.7-5.6)	4.9 (4.2-5.6)	4.9 (4.2-5.7)	4.2 (3.6-5.0)	4.5 (3.7-5.3)	<0.001
TG (mmol/L)	1.3 (0.9-1.8)	1.6 (1.0-2.4)	2.6 (1.7-4.1)	1.3 (1.0-2.0)	1.3 (0.9-1.9)	<0.001
eGFR (mL/min/1.73 m^2^)	100 (76-117)	106 (92-119)	113 (90-129)	91 (65-106)	94 (72-107)	<0.001
Hypertension, n (%)	22 (43.1)	108 (29.3)	91 (39.7)	158 (55.6)	189 (47.3)	<0.001
Coronary heart disease, n (%)	6 (11.8)	21 (5.7)	6 (2.6)	19 (6.7)	36 (9.0)	0.015
Cerebral vascular disease, n (%)	1 (2.0)	17 (4.6)	20 (8.7)	24 (8.5)	57 (14.3)	<0.001
DKD, n (%)	18 (35.3)	122 (33.2)	90 (39.3)	97 (34.2)	134 (33.5)	0.587
DR, n (%)	29 (56.9)	191 (51.9)	100 (43.7)	116 (40.8)	188 (47.0)	0.028
DPN, n (%)	49 (96.1)	334 (90.8)	173 (75.5)	235 (82.7)	346 (86.5)	<0.001
Types of diabetes therapy, n (%)						
Diet alone	3 (5.9)	1 (0.3)	2 (0.9)	21 (7.4)	15 (3.8)	<0.001
Non-insulin hypoglycemic agents	20 (39.2)	152 (41.3)	157 (68.6)	218 (76.8)	246 (61.5)	
Insulin	28 (54.9)	215 (58.4)	70 (30.6)	45 (15.8)	139 (34.8)	

Data are presented as median (25–75th percentile), or n (%). Chi-square test or Fisher’s test were used for between-group comparisons of categorical variables, and the Kruskal-Wallis test was used for analyses of non-normal distributions.

SAID, severe autoimmune diabetes; SIDD, severe insulin-deficient diabetes; SIRD, severe insulin-resistant diabetes; MOD, mild obesity-related diabetes; MARD, mild age-related diabetes; BMI, body mass index; SBP, Systolic Blood Pressure; DBP, Diastolic Blood Pressure; FPG, fasting plasma glucose; HbA1c, glycosylated hemoglobin; HOMA2-B, homeostatic model assessment 2 estimates of β-cell function; HOMA2-IR, homeostatic model assessment 2 estimates of insulin resistance; LDL-c, low density lipoprotein cholesterol; HDL-c, high density lipoprotein cholesterol; TC, total cholesterol; TG, triglycerides; eGFR, estimated glomerular filtration rate; DKD, diabetic kidney disease; DR, diabetic retinopathy; DPN, diabetic peripheral neuropathy.

There were significant differences in the lipid levels (all *P* < 0.001) among the five groups. Among them, the LDL-c, TC, and TG levels were higher in the SIDD and SIRD groups than in the other groups, whereas SAID had the highest HDL-c.

Regarding the diabetes-related complications, among the 5 clusters, the MOD group had the highest incidence of hypertension (55.6%); the MARD group, the highest incidence of CVD (14.3%); and the SAID group, the highest incidence of CHD (11.8%), DR (56.9%), and DPN (96.1%). there were significant differences among the five groups (all *P <*0.05). The SIRD group had the highest incidence of DKD (39.3%), which did not significantly differ from those of the other clusters (*P* = 0.587).

There were significant differences among the five groups with respect to diabetes treatment (*P* < 0.001). Compared with other clusters, the proportion of MOD patients receiving diet-only therapy was the highest (7.4%); the proportion of MOD patients receiving non-insulin hypoglycemic agents was the highest (76.8%), followed by SIRD patients (68.6%); the proportion of SIDD receiving insulin therapy was the highest (58.4%), followed by SAID (54.9%).

### Evaluation of diabetes treatment among clusters

**Figure 3:**
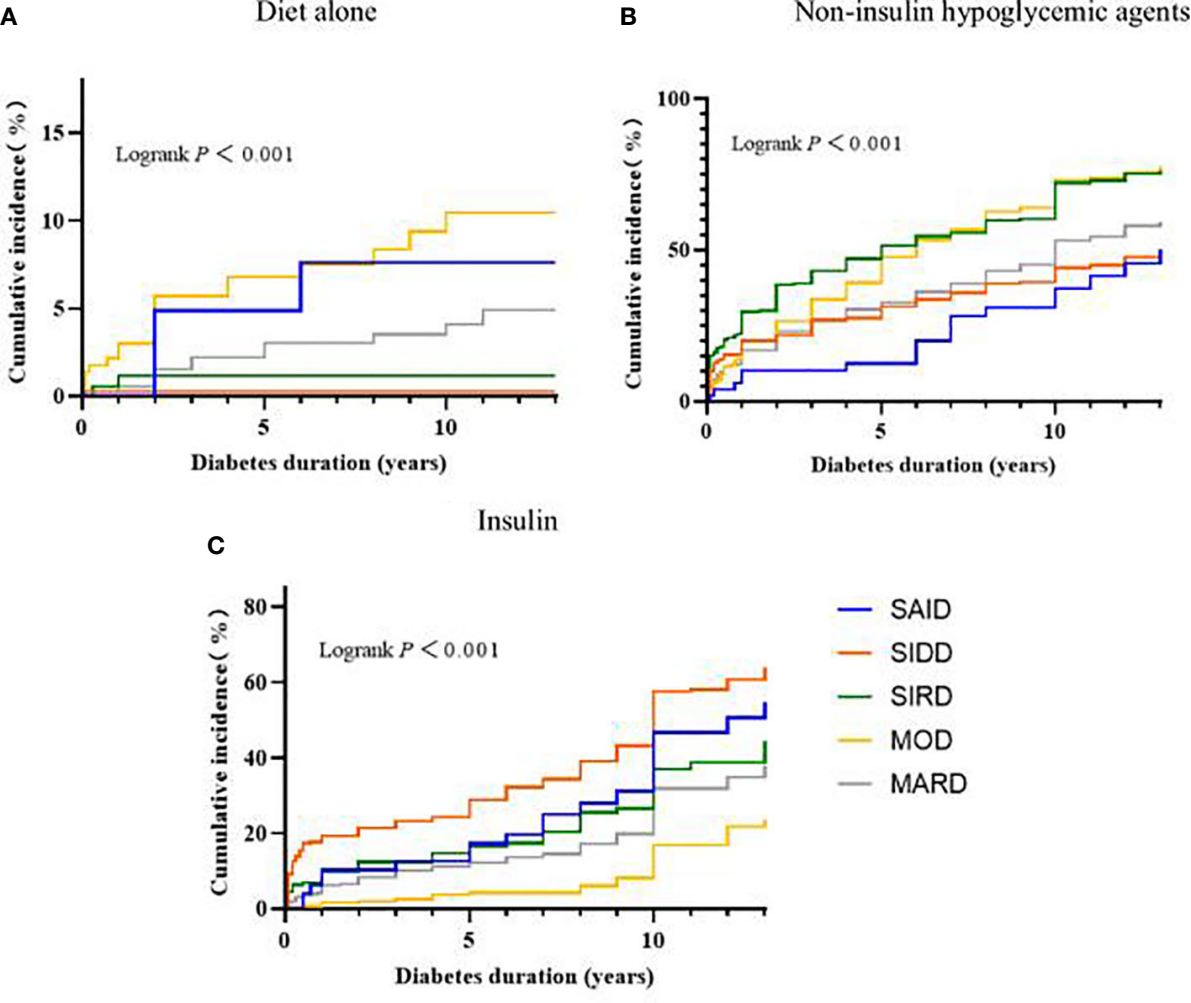
Progress in the treatment of diabetes between subgroups. Kaplan-Meier curves for the development of **(A)** Diet alone, **(B)** Non-insulin hypoglycemic agents, **(C)** Insulin. SAID, severe autoimmune diabetes; SIDD, severe insulin-deficient diabetes; SIRD, severe insulin-resistant diabetes; MOD, mild obesity-related diabetes; MARD, mild age-related diabetes.

The results of the Kaplan-Meier survival analysis suggested that, as the disease progressed, the patients with MOD received diet-only therapy the highest compared with the other clusters (Logrank test *P* < 0.001). The patients with SIRD and MOD received non-insulin hypoglycemic agents higher than other clusters (Logrank test *P* < 0.001). The SIDD group received insulin treatment higher than other clusters (Logrank test *P* < 0.001) (**Figure 3**).

According to Kaplan-Meier survival analysis, it can be seen that some cluster treatments will change after the duration of diabetes > 5 years. In addition, patients with a clinical course of diabetes > 5 years will gradually develop diabetes-related complications or other pathological changes that affect treatment. Therefore, hazard ratios for hypoglycemic treatment modalities in the subgroups were further compared and stratified by diabetes duration. The associations between the odds of receiving non-insulin hypoglycemic agents between subgroups are shown in **Table 2**. When the diabetes duration was ≤5 years, compared with SAID and SIDD, SIRD, MARD, and MOD have greater odds of receiving non-insulin hypoglycemic drugs (all *P* < 0.01), and the risk trends vary, with the variation of MOD being the most significant. When the duration of disease was >5 years, compared with the other subgroups, only the MOD group has greater odds of receiving non-insulin hypoglycemic agents (*P* < 0.01).

**Table 2:** Odds ratio of Non-insulin hypoglycemic agents (vs diet only or insulin) for each diabetes subgroup.

Diabetes subgroup					
	SAID	SIDD	SIRD	MARD	MOD
Diabetes duration ≤5 years					
Non-insulin hypoglycemic agents, n (%)	6 (1.3)	100 (21.5)	108 (23.2)	123 (26.5)	128 (27.5)
Odds ratio of Non-insulin hypoglycemic agents (95% CI)					
Crude model	reference.	1.79 (0.63-5.13)	5.81 (1.96-17.24) **	4.10 (1.42-11.88) **	8.53 (2.84-25.61) ***
Age‐ and sex‐adjusted model	reference.	1.74 (0.61-4.98)	5.75 (1.93-17.14) **	3.93 (1.33-11.58) *	8.32 (2.75-25.14) ***
Multivariable‐adjusted model†	reference.	2.00 (0.68-5.83)	6.70 (2.18-20.62) **	4.39 (1.46-13.20) **	9.11 (2.95-28.17) ***
Diabetes duration > 5 years					
Non-insulin hypoglycemic agents, n (%)	14 (4.3)	52 (15.9)	49 (14.9)	123 (37.5)	90 (27.4)
Odds ratio of Non-insulin hypoglycemic agents (95% CI).					
Crude model	reference.	0.63 (0.30-1.34)	1.79 (0.81-3.96)	1.77 (0.86-3.66)	3.29 (1.52-7.12) **
Age‐ and sex‐adjusted model	reference.	0.60 (0.28-1.27)	1.56 (0.70-3.49)	2.05 (0.98-4.28)	3.57 (1.64-7.77) **
Multivariable‐adjusted model†	reference.	0.62 (0.28-1.38)	1.96 (0.82-4.65)	1.96 (0.89-4.33)	3.65 (1.59-8.42) **

*P < 0.05, **P < 0.01, ***P < 0.001

†Adjusted for sex, age, diabetes duration, family history of diabetes, smoking, drinking, systolic blood pressure, diastolic blood pressure, diabetic retinopathy, diabetic peripheral neuropathy, diabetic kidney disease, Cerebral vascular disease, Coronary heart disease, and hypertension. SAID, severe autoimmune diabetes; SIDD, severe insulin-deficient diabetes; SIRD, severe insulin-resistant diabetes; MOD, mild obesity-related diabetes; MARD, mild age-related diabetes; CI, confident intervals.

The association between the odds of receiving insulin treatment between subgroups is shown in **Table 3**. Compared with MOD, SAID, SIDD, SIRD, and MARD have greater odds of receiving insulin (all *P* < 0.01) with different risk trends, with SAID having the most significant risk trend when the duration of diabetes was ≤5 years, and SIDD having the most significant risk trend when the duration of diabetes was >5 years.

**Table 3:** Odds ratio of insulin (vs diet only or non-insulin hypoglycemic agents) for each diabetes subgroup.

Diabetes subgroup					
	SAID	SIDD	SIRD	MARD	MOD
Diabetes Duration ≤5 years					
insulin, n (%)	8 (4.5)	92 (51.4)	29 (16.2)	41 (22.9)	9 (5.0)
Odds ratio of insulin (95% CI)					
Crude model	16.00 (4.87-52.54) **	14.57 (7.02-30.25) **	4.22 (1.92-9.28) **	4.97 (2.33-10.62) **	reference.
Age‐ and sex‐adjusted model	15.54 (4.70-51.36) **	14.16 (6.79-29.53) **	3.95 (1.74-8.93) *	5.07 (2.36-10.87) **	reference.
Multivariable‐adjusted model†	17.49 (5.03-60.82) **	13.34 (6.16-28.87) **	4.73 (1.92-11.69) *	4.87 (2.20-10.82) **	reference.
Diabetes Duration > 5 years					
insulin, n (%)	20 (6.3)	123 (38.7)	41 (12.9)	98 (30.8)	36 (11.3)
Odds ratio of insulin (95% CI)					
Crude model	3.52 (1.63-7.61) *	6.24 (3.78-10.31) **	2.21 (1.26-3.89) *	2.01 (1.26-3.19) *	reference.
Age‐ and sex‐adjusted model	3.78 (1.74-8.24) *	7.11 (4.24-11.94) **	2.66 (1.48-4.79) *	1.90 (1.19-3.04) *	reference.
Multivariable‐adjusted model†	3.91 (1.68-9.10) *	7.19 (4.05-12.76) **	2.44 (1.25-4.77) *	2.04 (1.23-3.39) *	reference.

*P < 0.01, **P < 0.001.

†Adjusted for sex, age, diabetes duration, family history of diabetes, smoking, drinking, systolic blood pressure, diastolic blood pressure, diabetic retinopathy, diabetic peripheral neuropathy, diabetic kidney disease, Cerebral vascular disease, coronary artery disease, hypertension. SAID, severe autoimmune diabetes; SIDD, severe insulin-deficient diabetes; SIRD, severe insulin-resistant diabetes; MOD, mild obesity-related diabetes; MARD, mild age-related diabetes; CI, confident intervals.

### Application characteristics of diabetes drugs among clusters

The application of nine hypoglycemic drugs among the diabetes clusters is shown in detail in **Table 4**. When the duration of diabetes is ≤5 years, compared with the other subgroups, the SAID and SIDD groups had higher usage rates of metformin, AGI, and insulin; the SIRD group had higher usage rates of metformin, AGI, and GLP-1RA; the MOD and MARD groups had higher usage rates of metformin, AGI, and SGLT-2i. When the duration of diabetes was >5 years, the choice of insulin among the clusters generally increased, as compared with the duration of ≤5 years, although the choice of non-insulin hypoglycemic agents was similar between the two durations.

**Table 4:** Characteristics of medication application in diabetes subgroups.

	SU, N (%)	Glinides, N (%)	Metformin, N (%)	TZDs, N (%)	AGI, N (%)	DPP-4, N (%)	SGLT-2i, N (%)	GLP-1RA, N (%)	Insulin, N (%)
Diabetes duration ≤ 5 years									
SAID (n=16)	0 (0.0)	0 (0.0)	8 (50.0)	1 (6.3)	9 (56.3)	1 (6.3)	4 (25.0)	3 (18.8)	8 (50.0)
SIDD (n=193)	9 (4.7)	12 (6.2)	120 (62.2)	80 (41.5)	135 (69.9)	16 (8.3)	69 (35.8)	46 (23.8)	92 (47.7)
SIRD (n=139)	5 (3.6)	4 (2.9)	98 (70.5)	61 (43.9)	67 (48.2)	7 (5.0)	45 (32.4)	80 (57.6)	29 (20.9)
MOD (n=153)	12 (7.8)	11 (7.2)	53 (34.6)	31 (20.3)	54 (35.3)	27 (17.6)	47 (30.7)	14 (9.2)	9 (5.9)
MARD (n=173)	11 (6.4)	17 (9.8)	75 (43.4)	43 (24.9)	102 (59.0)	35 (20.2)	44 (25.4)	9 (5.2)	41 (23.7)
Diabetes duration > 5 years									
SAID (n=35)	2 (5.7)	2 (5.7)	13 (37.1)	8 (22.9)	26 (74.3)	6 (17.1)	9 (25.7)	4 (11.4)	20 (57.1)
SIDD (n=175)	12 (6.9)	21 (12.0)	79 (45.1)	41 (23.4)	128 (73.1)	14 (8.0)	70 (40.0)	37 (21.1)	123 (70.3)
SIRD (n=90)	7 (7.8)	6 (6.7)	49 (54.4)	31 (34.4)	46 (51.1)	16 (17.8)	42 (46.7)	42 (46.7)	41 (45.6)
MOD (n=131)	10 (7.6)	18 (13.7)	49 (37.4)	30 (22.9)	71 (54.2)	22 (16.8)	42 (32.1)	18 (13.7)	36 (27.5)
MARD (n=227)	20 (8.8)	18 (7.9)	86 (37.9)	47 (20.7)	148 (65.2)	30 (13.2)	77 (33.9)	30 (13.2)	98 (43.2)

All values were N (%). SU, sulphonylurea; TZDs, thiazolidinediones; AGI, α-glucosidase inhibitor; DPP4-I, dipeptidyl peptidase 4 inhibitors; SGLT2i, sodium-glucose co-transporter 2 inhibitors; GLP-1RA, glucagon-like peptide-1 receptor agonists; SAID, severe autoimmune diabetes; SIDD, severe insulin-deficient diabetes; SIRD, severe insulin-resistant diabetes; MOD, mild obesity-related diabetes; MARD, mild age-related diabetes.

## Discussion

The present retrospective study clarified the clustering-based diabetes subgroups of adult-onset diabetes in a Chinese population. To the best of our knowledge, this is the first study to date that found that the distribution of diabetes clusters in the Chinese population is consistent with the five clusters (SAID, SIDD, SIRD, MOD, and MARD) reported by Ahlqvist et al. (4). The purpose of subdividing and clustering patients with diabetes is to promptly identify groups with different characteristics and to accurately treat them. Thus, this study not only analyzed the differences in diabetes-related complications among clusters as in previous studies (5, 6) but also focused on the treatment of patients in each cluster as the course of diabetes progressed for the first time. We found significant differences in the treatment modalities among the five diabetes clusters. Moreover, the treatment modalities were associated with disease duration. Early in the course of disease (≤5 years), compared with the other subgroups, the SIRD, MOD, and MARD populations were more likely to receive non-insulin hypoglycemic agents for glycemic control. Among the non-insulin hypoglycemic drug options, SIRD had higher rates of receiving metformin, AGI, and GLP-1RA drugs; the MOD and MARD groups both received metformin, AGI and SGLT-2i drug ratio was higher. While the SAID and SIDD groups were more likely to receive insulin therapy than the other subgroups. However, with prolonged disease course (>5 years), only the MOD population can receive non-insulin hypoglycemic drugs to control blood sugar, and most of these patients still receive treatment with metformin, AGI, and SGLT-2i drugs, whereas the other four groups need to receive insulin therapy.

By using the Two-Step clustering method established by Ahlqvist et al. (4), we replicated the five subgroups of adult-onset diabetes in Chinese patients. The SAID cluster was GADA-positive and had a younger age of onset; the SIDD cluster had severe insulin deficiency and the highest HbA1c; the SIRD cluster had severe insulin resistance and high BMI; and the MOD cluster had a higher BMI and was slightly younger than the MARD cluster. These results are similar to previous studies involving Japanese (6), Chinese and American populations (8, 12). The reproducibility among different ethnic groups suggests that this method is applicable to the Chinese population. However, there are also differences. In our and other reported studies of East Asians including the Chinese (5, 6, 8, 13), the BMI of the MOD cluster is generally lower than that of Caucasians (4, 7), which may be closely related to the dietary patterns of the different ethnic groups. The Chinese diet is characterized by a high intake of whole grains and vegetables, and a higher intake of fiber, thus reducing the risk of obesity (14). Second, HOMA2-IR and age at diagnosis were significantly lower in the SIRD clusters than in Caucasians (4, 7), the Japanese SIRD cluster (6) also showed the same trend. A previous study has reported that East Asians have lower insulin secretion and insulin resistance than Caucasians (15). Overall, this partially explains the differences in the frequency of SAID, SIDD, and SIRD clusters between Chinese and Caucasians. It also showed that East Asians with a low endogenous insulin secretion capacity are more susceptible to the development of diabetes in a state of overnutrition; thus, they develop diabetes at a younger age.

Our study found that the SAID clusters exhibited a higher risk of DR, DPN, and CHD, which was somewhat consistent with Tanabe et al. (6). Type 1 diabetes (SAID in the current study) is known to be associated with an increased risk of cardiovascular disease and microvascular complications (16, 17), the main mechanism of which has not been fully clarified, but it may be partly related to the high levels of metalloproteinase-9 and metalloproteinase-1 tissue inhibitors in the plasma and tissues of patients with type 1 diabetes (18). The SIRD group has the highest incidence of DKD, which is consistent with the finding of previous reports (8, 19) and may be related to the high smoking rate and lipid metabolism disorder in patients with SIRD. Studies have found that smoking and abnormal serum lipids are important factors that promote the development of DKD (20, 21); in addition, obese patients with SIRD subgroups may develop chronic kidney disease into obesity-related glomerulopathy rather than typical DKD (22). The patients with MARD have the highest incidence of CVD, and it is well known that elderly patients with diabetes have been the key population for the prevention of CVD (23).

From the survival analysis curve, the prevalence of antidiabetic drug therapy increases with the duration of diabetes, which is consistent with the results of a previous study (24). Analyzing the choice of hypoglycemic agents among each cluster, the choice of insulin in each cluster generally increased with the prolongation of diabetes duration. The progression from normal glucose tolerance to diabetes in patients with diabetes is characterized by a decrease in islet β-cell mass or accompanied by insulin resistance (25–27), leading to excessive and prolonged elevation of glucose levels and the resulting glucotoxicity that further leads to β-cell dysfunction and apoptosis, affecting β-cell proliferation (28, 29); in addition, with prolonged disease course, the high glucose level state in patients with diabetes interacts with cell senescence, forming a “malignant positive feedback”, which further promotes insulin resistance and pancreatic β-cell dysfunction (30). Therefore, patients with long-term diabetes, especially those with poor glycemic control, although treatment with non-insulin hypoglycemic agents increased insulin sensitivity and promoted insulin secretion, endogenous insulin still encountered difficulty in meeting the hypoglycemic needs, and hence should be supplemented with exogenous insulin.

Although the demand for insulin will increase in the later stages of diabetes, in our study, we found that there were significant differences in the treatment of each diabetes cluster at different stages of diabetes. In clinical setting, we will select more appropriate hypoglycemic drugs according to whether the patient has diabetes-related complications, so the effect of diabetes-related complications was excluded in the multivariate regression analysis that analyzed the relationship between each cluster and diabetes treatment. We found that patients with SIDD and SAID were treated in a similar manner throughout the diabetes period, with a significant need for insulin therapy, as demonstrated in previous studies (4, 8). This is also consistent with the pathological characteristics of these two types of patients. SAID is equivalent to the type 1 diabetes population in the traditional classification and is characterized by severe insulin deficiency caused by autoimmunity-induced damage to the pancreatic β cells (27), and SIDD is a cluster characterized by severe insulin deficiency in the type 2 diabetes population. The other three type 2 diabetes clusters (SIRD, MARD and MOD) showed different treatment trends. In the early stage of diabetes (≤5 years), patients with SIRD, MARD, and MOD can meet their hypoglycemic needs with non-insulin drugs. Whereas, in the later stage of diabetes (>5 years), patients with SIRD and MARD need additional insulin therapy, only the MOD population can be treated with non-insulin drugs to meet glycemic needs. Although the MOD population has higher BMI and insulin resistance, the islet function of the MOD population was the best among the five clusters; thus, the insulin secreted through the excellent islet function can meet the insulin demand in the body under insulin resistance. However, in patients with SIRD and MARD, the islet secretion function *in vivo* cannot overcome the negative effects of insulin resistance or cell senescence on islet function with the prolongation of the course of the disease. This may be the reason for the difference in hypoglycemic therapy among these three groups. We also analyzed the selection of various hypoglycemic drugs among the clusters in different periods. The use of hypoglycemic agents in each cluster is in line with the recommendations of the American Diabetes Association and Chinese diabetes guidelines for metformin as the first-line drug for the treatment of type 2 diabetes (9, 31); thus, metformin usage was higher in each type 2 diabetes cluster. The application of AGI was also higher in all clusters in our study population, which may be related to the faster gastric emptying time in Chinese patients, resulting in greater fluctuation of postprandial blood glucose levels (32); thus, AGI is needed to regulate the postprandial blood sugar level. GLP-1RA has a significant effect on weight loss, can increase insulin secretion and slow the progress of DKD (33, 34); thus, it is more suitable for the SIRD population with obesity, high insulin resistance and high incidence of DKD. SGLT-2i is beneficial for weight loss, increases insulin sensitivity, lower blood pressure, has cardiovascular and cerebrovascular protective effects, and has a low risk of hypoglycemia (35–37); in addition, SGLT-2i can induce the conversion of glucosuria to a state of systemic energy deficit and attenuate cellular senescence as a caloric restriction mimetic (37); thus, it is suitable for MOD and MARD populations who are older, overweight, and at high risk of hypertension and CVD.

This study has some limitations. First, this is a retrospective study; therefore, the current analysis cannot draw conclusions about the causal relationship between the various clusters of diabetes and the type of diabetes treatment. Second, the participants in this study were small, as compared to the Swedish study; thus, future confirmatory studies are needed. Third, this is a single-center study, and all the study participants were inpatients, so the enrolled patients had limited expansion to the other populations. Finally, the current cluster classification of diabetes can only be statistically subdivided by the existing clinical indicators of patients with diabetes, as there is no specific parameter cut-off value to directly classify new patients with diabetes. To extend our research and comprehensively assist the clinical treatment of diabetes, more research is needed in the future to define a decision support system.

## Conclusions

This study demonstrated that clustering of patients with diabetes with a data-driven approach can yield consistent results. Each diabetes cluster has significantly different disease characteristics and risk of diabetes complications. With the development of the disease course, each cluster receives different hypoglycemic treatments. This finding provides an important basis for the etiological stratification and individualized management of different clusters of diabetes.

## Data availability statement

The raw data supporting the conclusions of this article will be made available by the authors, without undue reservation.

## Ethics statement

Our study protocol had been approved by the ethics committee of the First Affiliated Hospital of Nanchang University (No. 2022-4-025), and the ethics committee has approved the use of anonymized historic data in the study and waived informed consent from patients.

## Author contributions

JZ performed data analysis, interpretation and manuscript writing. JZ and YD supervised the data collection and research collaboration. JZ, YD, YW and JW participated in data collection and literature search. JZ and JX designed the experiment and supervised the overall progress. All authors contributed to the article and approved the submitted version.

## Funding

This study was supported by grants from the National Natural Science Funds of China (grant no. 81760168).

## Conflict of interest

The authors declare that the research was conducted in the absence of any commercial or financial relationships that could be construed as a potential conflict of interest.

## Publisher’s note

All claims expressed in this article are solely those of the authors and do not necessarily represent those of their affiliated organizations, or those of the publisher, the editors and the reviewers. Any product that may be evaluated in this article, or claim that may be made by its manufacturer, is not guaranteed or endorsed by the publisher.
