# Supplementary material for: Diabetes duration and types of diabetes treatment in data-driven clusters of patients with diabetes
Source: Front Endocrinol (Lausanne). 2022 Nov 15;13:994836. doi: 10.3389/fendo.2022.994836 (PMC9705576; doi:10.3389/fendo.2022.994836)
Supplement: Supplementary file 1 [file Table_1.doc]

Table S1 Cluster centers of five classifications in Chinese population with diabetes mellitus

| **Cluster** | **HbA_1C_** | **BMI** | **Age** | **HOMA2-B** | **HOMA2-IR** |
| --- | --- | --- | --- | --- | --- |
| 1/SAID | 9.1±2.2 | 23.2±3.2 | 45.9±11.4 | 52.2±42.3 | 1.6±0.8 |
| 2/SIDD | 11.5±1.9 | 22.9±2.7 | 45.5±11.8 | 26.8±15.1 | 1.6±0.7 |
| 3/SIRD | 9.9±1.8 | 29.0±3.4 | 39.5±11.6 | 49.1±28.5 | 3.2±1.5 |
| 4/MOD | 6.6±1.0 | 24.9±3.5 | 50.8±11.7 | 134.8±40.3 | 2.2±0.9 |
| 5/MARD | 7.8±1.2 | 23.1±2.6 | 53.7±10.2 | 57.7±22.9 | 1.7±0.7 |

SAID, severe autoimmune diabetes; SIDD, severe insulin-deficient diabetes; SIRD, severe insulin-resistant diabetes; MOD, mild obesity-related diabetes; MARD, mild age-related diabetes; HbA1c, glycosylated hemoglobin; BMI, body mass index; HOMA2-B, homeostatic model assessment 2 estimates of β-cell function; HOMA2-IR, homeostatic model assessment 2 estimates of insulin resistance.
